# Advanced fibrosis, *TP53* variants and acute/pure erythroid leukaemia: Diagnostic harbingers of *ERCC6L2* ‐related bone marrow failure syndrome?

**DOI:** 10.1111/bjh.70666

**Published:** 2026-07-07

**Authors:** Sai Sudha Valisekka, Meghan E. Lindstrom, Olivia J. Michel, Roy L. Kao

**Affiliations:** ^1^ Department of Medicine University of Minnesota Minneapolis Minnesota USA; ^2^ Department of Laboratory Medicine and Pathology University of Minnesota Minneapolis Minnesota USA; ^3^ Cancer Risk Management Program, Fairview Health Services Minneapolis Minnesota USA; ^4^ Division of Hematology, Oncology and Transplantation, Department of Medicine University of Minnesota Minneapolis Minnesota USA

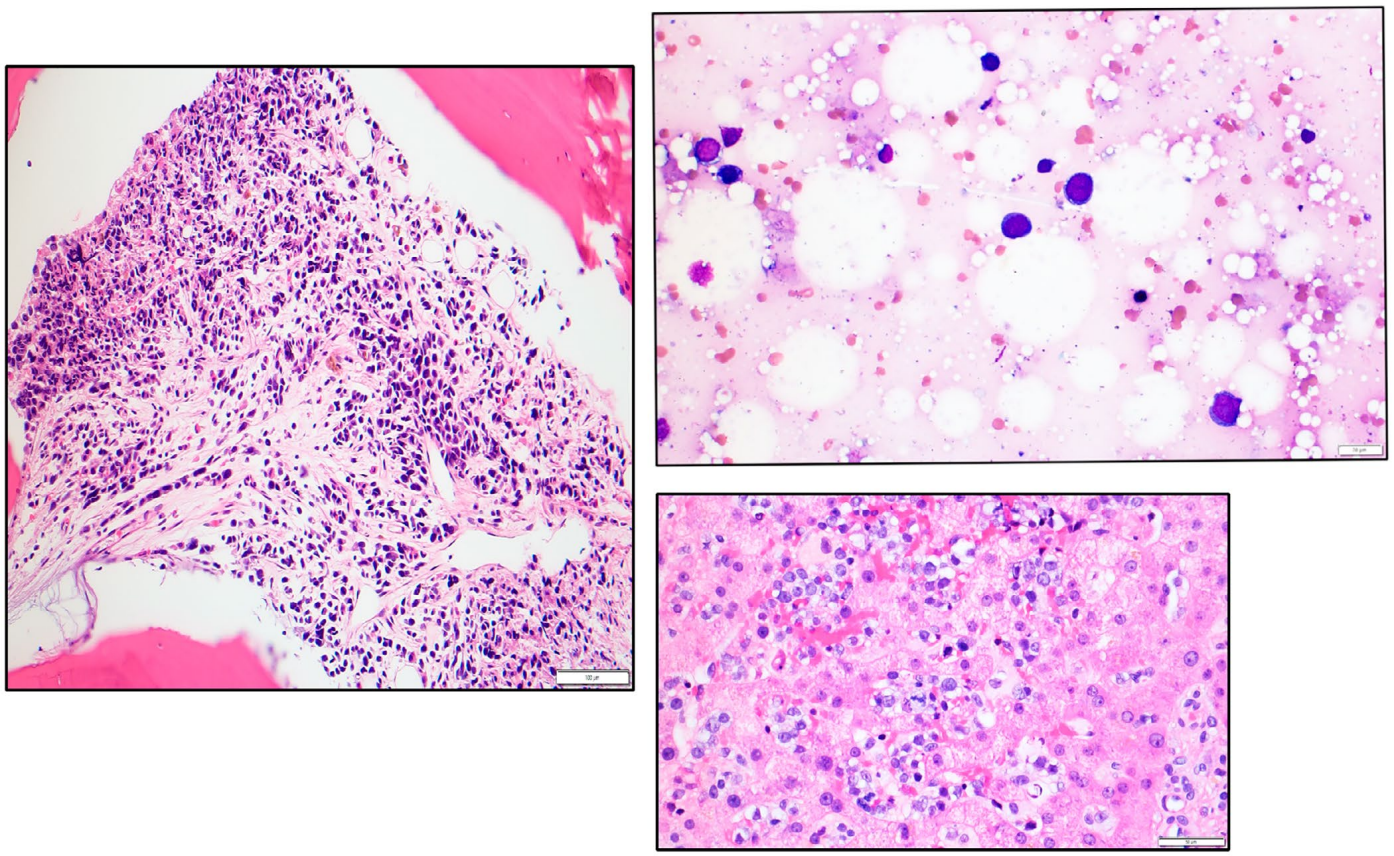

A 27‐year‐old male presented with neutrophil count 0.8 × 10^9^/L (reference range: 1.6–8.1), haemoglobin 39 g/L (131–171), platelets 19 × 10^9^/L (179–450) and fever. Bone marrow (BM) biopsy revealed patchy sheets of large, atypical cells with round to slightly irregular nuclear contours; delicate, open chromatin with inconspicuous nucleoli; and moderate to scant eosinophilic cytoplasm (left, haematoxylin and eosin [H&E], objective ×10). These stained positive for E‐cadherin and cluster of differentiation (CD)‐43, consistent with erythroid precursors (not shown), and diffuse, strong nuclear p53 expression (not shown). European Consensus grade 3 out of 3 reticulin fibrosis (not shown) precluded precise evaluation of cellularity. Touch imprints (top right, H&E, objective ×50), nearly acellular, showed cells with ovoid nuclei, fine chromatin with prominent nucleoli and scant, deeply basophilic cytoplasm, suggestive of atypical pronormoblasts. Failed cell culture precluded cytogenetic evaluation. Next‐generation sequencing identified three pathogenic tumor protein p53 (*TP53*) variants. Germline genetic testing on cultured skin fibroblasts revealed a pathogenic homozygous exon 11 deletion in the excision repair cross‐complementing 6 like 2 gene (*ERCC6L2*), establishing a diagnosis of autosomal recessive *ERCC6L2*‐related bone marrow failure syndrome.

Despite chemotherapy, he developed a new liver mass. Biopsy showed diffuse predominantly sinusoidal infiltration by large atypical cells with scant cytoplasm and large nuclei containing vesicular chromatin and prominent central nucleoli (bottom right, H&E, objective ×20). These demonstrated dim CD45, patchy E‐cadherin, strong CD43 and diffuse, strong CD117 and glucose transporter 1 (GLUT1) expression (not shown), consistent with myeloid blasts with erythroid differentiation (erythroblasts). Diffuse, strong nuclear p53 expression was also present (not shown). Overall, findings were consistent with myeloid sarcoma, arising from acute/pure erythroid leukaemia (AEL/PEL). He eventually died of disease relapse involving BM, liver and pleura despite high‐dose cytarabine followed by myeloablative peripheral blood haematopoietic stem cell transplantation from an human leukocyte antigen‐matched sibling, a heterozygous carrier for the same *ERCC6L2* variant. Genetic counselling for family members was provided.

AEL/PEL in this patient supports emerging evidence that *ERCC6L2*‐related leukaemias show an association with the erythroid lineage.^1^ This case also demonstrates the possibility of extra‐medullary involvement, including liver and pleural infiltration. The initial early erythroid precursors seen on BM biopsy likely represented blast equivalents for this patient. Together, advanced reticulin fibrosis, erythroid predominance and *TP53* mutation may represent a characteristic pathological triad in *ERCC6L2*‐related disease.^2^

## FUNDING INFORMATION

The author(s) received no financial support for the research, authorship and/or publication of this article.

## ETHICS STATEMENT

At University of Minnesota, this individual case report does not meet the definition of Human Research as it does not meet the ‘systematic investigation’ or ‘generalizable knowledge’ thresholds. Therefore, this case report did not require IRB review.

## PATIENT CONSENT

Written informed consent was obtained from the next of kin of the deceased patient for publication of this case report and any accompanying images.
